# Body Mass Analysis for Patients Undergoing Surgical Intervention: A Cross‐Sectional Study

**DOI:** 10.1002/hsr2.71707

**Published:** 2025-12-29

**Authors:** Sazgar Khald Ibrahim, Rondik Khalid Mosa, Alaa Noori Sarkees, Salih M. S. Zebari

**Affiliations:** ^1^ Department of General Surgery, College of Medicine Hawler Medical University (HMU) Erbil Iraq; ^2^ Duhok Obstetrics and Gynecology Hospital Duhok Iraq; ^3^ Department of Nursing, College of Health and Medical Technology Duhok Polytechnic University (DPU) Duhok Iraq; ^4^ Department of Animal Resource, College of Agricultural Engineering Sciences Salahaddin University‐Erbil Erbil Iraq; ^5^ Department of Nutrition and Dietetics Cihan University‐Erbil Erbil Iraq

**Keywords:** BIA, Body mass analysis, Erbil city, surgical intervention

## Abstract

**Background and Aim:**

Bioelectrical Impedance Analysis is a reliable, noninvasive, objective, and cost‐effective body composition assessment method used in clinical practice. Body composition provides information for precise nutrition intervention in the perioperative period to improve patients' postoperative outcomes. The study evaluates and estimates the nutritional status of patients undergoing surgical intervention.

**Patients and Methodology:**

A cross‐sectional survey took place from March 2024 to October 2024 to examine the body composition, muscle‐fat analysis, and body‐calculated data of 2368 adult patients (aged 18 years and older) from Erbil Teaching Hospital in Erbil City utilizing a convenience sampling technique from those patients who were admitted to the hospital for surgical intervention. Data were collected using the InBody machine 270, including two parts: the first was related to demographic data, while the second consisted of body composition.

**Results:**

The analysis showed that men generally had higher levels of total body water, protein, minerals, skeletal muscle mass and fat‐free mass, while women indicated higher levels of visceral fat and percent body fat, as both genders show waist‐hip ratios and levels of obesity above the average.

**Conclusion:**

The study concluded that gender differences have a significant impact on the presence of gender body composition differences.

## 1 Introduction

Nowadays the primary treatment for many solid tumors is surgery. Overall survival and quality of life have increased significantly as a result of advancements in surgical procedures over the past 20 years [1]. However, if systemic issues are not considered, surgery cannot have a meaningful impact. For example, it has been shown that postoperative complications and hospital stay duration are significantly influenced by nutritional status [2].

Malnutrition has long been known to be a contributing factor in mortality, post‐operative complications, extended hospital stays, and ultimately, increased healthcare expenses. Furthermore, malnutrition is frequently linked to pathologic conditions that raise the risk of surgery, such as cancer, chronic inflammation, or damage to organs [3]. One of the independent variables that most likely affects the post‐operative outcomes of elective procedures is the patient's nutritional status. The organic reaction to surgical trauma has more consequences and adversely affects the outcome in patients who are malnourished or at risk of malnutrition [4]. Establishing nutritional monitoring practices is crucial in order to detect those who are malnourished or at risk of malnutrition early on and start pre‐operative nutritional therapy (NT) [5]. The goal of enhanced recovery programs is to decrease hospital stays by improving post‐operative recovery and lowering overall morbidity following surgery. These programs include a number of perioperative measures, most of which are evidence‐based. In fact, multimodal care that incorporates physical exercise and nutritional support when necessary is strongly advised. One of the main interventions of the current improved recovery after surgery (ERAS) program is the early consumption of oral foods [3]. Nevertheless, the current enhanced recovery after surgery guidelines either advocate ad‐lib fluids or do not specify the amount of postoperative oral fluids or food items [6]. Patients who are malnourished or at nutritional risk should all receive nutrition education and nutrition support five‐step therapy, according to the European Society for Clinical Nutrition and Metabolism guidelines [7, 8]. The Subjective Global Assessment (SGA) is a validated method for evaluating nutritional status that is linked to clinical outcomes [9, 10, 11].

Healthcare practitioners do not routinely utilize it to evaluate nutritional decline in hospitalized patients, with the exception of Braunschweig's study. Although it is more frequently recorded on the wards, body weight has limitations because of fluid status variations. Extended length of stay (LOS) has been linked to a 5% weight loss, excluding patients with fluid problems [12]. Numerous approaches to nutritional evaluation (NE) have been brought up, including physical composition tests, biochemical, anthropometric, and clinical evaluation tests. In general, anthropometric techniques are easy to use, straightforward, non‐invasive, and cost‐free. They also increase the precision and accuracy of nutritional disease diagnosis when combined with other objective criteria (e.g., laboratory) [13]. Bioelectrical Impedance Analysis (BIA) is frequently used in clinical practice to assess body composition because it is a dependable, non‐invasive, objective, cost‐effective, and time‐efficient approach [14]. Nevertheless, there are uncertainties in its estimations [15]. The purpose of the study is to ascertain the patient's preoperative body mass composition and how it relates to gender.

## 2 Patients and Methods

### 2.1 Study Design and Patients

A cross‐sectional survey is planned to take place from March to October 2024 in order to examine the body composition, muscle‐fat analysis, and body‐calculated data of 2368 adult patients undergoing surgical intervention.

### 2.2 Sample and Sampling

A convenience sampling technique was utilized to enroll a total of 2368 adult patients (aged 18 years and older) from Erbil Teaching Hospital in Erbil City between March to October 2024. Those patients who were admitted to the hospital for surgical intervention were systematically screened for inclusion in the current study.

### 2.3 Data Collection

Data in the present survey were collected using the same InBody machine 270 by the same interviewer including two parts, the first related to demographic data such as age, gender, and body mass index, while the second consisted of body composition as body water, protein, minerals, body fat mass, weight, skeletal muscle mass, fat‐free mass, visceral fat level, percent body fat, basal metabolic rate, Inbody score, waist‐hip ratio, obesity degree.

### 2.4 Inclusion Criteria

Patients were considered eligible for this study if they were at least 18 years old, had a planned length of hospital stay longer than 24 h, and agreed to be subject to this study.

### 2.5 Exclusion Criteria

The current study excludes; Pregnancy and critical illness, patients defined as the failure of one vital organ needing intensive care, all the patients considered as critical, terminal, or non‐terminal, whether or not in an ICU unit, and patients with known medical conditions that could impact body composition, such as severe endocrine disorders, to maintain the accuracy of the data.

### 2.6 Data Collection and Nutritional Status Variables or Anthropometry and Body Composition Measurement

Several nutritional status variables were taken using a standardized body composition analysis, which was performed using BIA for several anthropometric and other composition measurements with association of total body water, protein levels, mineral content, body fat mass, skeletal muscle mass, fat‐free mass, visceral fat level, The measurement was performed on preoperative day one before the surgery. To ensure measurement accuracy and repeatability, the analysis was performed in compliance with the manufacturer's instructions. Measurements Included: Body Mass Index (BMI). According to WHO criteria, BMI is used to classify individuals as Underweight < 18.5 Kg/M^2^, Normal weight 18.5 to < 25 kg/M^2^, Overweight 25 to < 30 kg/M^2^, and Obese 30 kg/M^2^ and more. Percent Body Fat is categorized as Low (< 10% for males and < 18% for females), Normal (10%–20% for males and 18%–28% for females), and High (> 20% for males and 28% for females). Basal Metabolic Rate (BMR) is classified as Low < 1600 Calories, Normal 1600–2000 Calories, and High > 2000 Calories. The InBody score reflects the overall health state, ranging from a health risk < 60 score, Low Risk 60 to < 70 score, Normal 70–80 score, and Athlete > 80 score. Visceral Fat Level measures the potential for excessive fat accumulation around internal organs. Healthy levels are 1–12, and the Risk of excessive levels ≥ 13. Waist–Hip Ratio (WHR) measure shows fat distribution patterns and can be Low (< 75 For Males and < 80 For Females), Normal (75–85 For Males and 80–90 For Females), and High (> 85 for Males and > 90 for Males). The degree of Obesity can be categorized as Below Range < 90, Normal Range 90–110, and Over Range > 110. Whereas, the rest of the parameters, researchers consider the individualized parameters' references and ranges, which are recommended by the In Body Machine results and recommendations for each patient, concerning their age and gender.

### 2.7 Participant Flow

All consecutive adult surgical admissions between March and October 2024 were screened for eligibility. Exclusions occurred for pregnancy, critical illness, terminal states, or severe endocrine disorders, while eligible patients who consented were included. A total of 2368 patients were enrolled and analyzed, with no further attrition.

### 2.8 Missing Data

Data completeness was verified prior to analysis. Missing values were minimal (< 2% across variables) and analyses were conducted using complete‐case data. Given the low proportion and random nature of missingness, imputation was not required.

### 2.9 Bias and Generalizability

Although consecutive screening reduced selection bias, the use of a single referral hospital and a predominantly female sample may limit external generalizability. These contextual factors are acknowledged, and findings should be interpreted as representative of surgical patients in Erbil City rather than the wider Iraqi or regional population.

### 2.10 Statistical Analysis

The data were entered and analyzed using the SPSS program version 23 IBM. Descriptive statistics such as frequency, percentage, mean, standard deviation (SD), and confidence intervals were calculated for subjects overall and within each BMI category to summarize the data. The association between gender and body composition was examined using Chi‐square tests, and linear Regression with a *p*‐value equal to or less than 0.05 indicating statistical significance.

### 2.11 Ethical Considerations

The study protocol was approved by the ethical committee of Hawler Medical University (6) on 8‐5‐2024. Written informed consent was obtained from each participant to ensure their privacy and the option to withdraw at any time. This study was conducted in accordance with the 1964 Helsinki Declaration.

## 3 Results

Table 1. indicates that most of the patients were female 1549 (65.4%) and ranged between the ages of 18 and 47 years.

**Table 1:** Distribution of the patients′ demographic variables.

Category		Frequency	Percent = F/2368
**Gender**	Female	1549	65.4
	Male	819	34.6
**Age**	18–27	705	29.8
	28–37	602	25.4
	38–47	622	26.3
	48–57	315	13.3
	58–67	92	3.9
	68+	32	1.4

Table 2 presents that the total body water of nearly half of men is normal (47.7%), followed by high (47.3%), while in women, it is high (49.6%), followed by normal (35.8%). Half (50.3%) of the males show a high protein level, followed by a normal level (45.5%), however, less than half of the females (47.8%) show high, followed by (38.1%). Also, approximately half of the males (49%) had normal mineral levels, followed by high (44.8%), while less than half of the females (46.2%) had high, followed by normal levels (45.5%). Moreover, most males and females reveal high levels of body fat mass at (85.6%) and (89.8%), respectively. Furthermore, the body compositions were significantly associated with patients' gender, except for minerals. At the same time, linear regression results show that male gender indicates a higher impact on all body composition than female, but a lower impact on body fat mass, at *p*‐value ≤ 0.05.

**Table 2:** Distribution of the patients by their body composition analysis.

		Male *N* (% = *N*/819)	Female *N* (% = *N*/1549)	*p* value
Total body water	Low	41 (5)	227 (14.7	≤ 0.001*
	Normal	391 (47.7)	554 (35.8)	
	High	387 (47.3)	768 (49.6)	
B confidence interval (L–U)		12.092 (11.503–12.680)		≤ 0.001**
Protein	Low	34 (4.2)	219 (15.1)	≤ 0.001*
	Normal	373 (45.5)	590 (38.1)	
	High	412 (50.3)	740 (47.8)	
B confidence interval (L–U)		3.358 (3.200–3.517)		≤ 0.001**
Minerals	Low	51 (6.2)	128 (8.3)	0.103*
	Normal	401 (49)	705 (45.5)	
	High	367 (44.8)	716 (46.2)	
B confidence interval (L–U)		1.092 (1.043–1.141)		≤ 0.001**
Body fat mass	Low	27 (3.3)	23 (1.5)	0.002*
	Normal	91 (11.1)	135 (8.7)	
	High	701 (85.6)	1391 (89.8)	
B confidence interval (L–U)		−4.470 (−6.045 to −2.895)		≤ 0.001**

* Chi‐Square.

** Linear Regression, *p* value at ≤ 0.05.

Table 3 reveals that more than half of the males (51%) had an over Skeletal Muscle Mass level, whereas less than half of the females (46.4%) had a normal level. At the same time, less than half of males and females show high levels of Fat‐Free Mass, 48.8% and 46.6%, respectively. Furthermore, the Visceral Fat Level stands for more than half (52.6%) as a healthy level among the males; on the other hand, the females show close to three quarters (71.1%) had a risk of excessive level of Visceral Fat. In addition, a large percentage of both males and females present a high percent of Body Fat, 88.3% and 99.4% respectively. Besides all, the above Muscle‐Fat variables show a significant association with the gender of the patients. Moreover, a linear regression reveals that male gender has a higher impact on body skeletal muscle mass and fat‐free mass than female, however, a lower impact on body Visceral Fat Level and percent body fat, at *p*‐value ≤ 0.05.

**Table 3:** Distribution of the patients by their Muscle–Fat analysis.

		Male *N* (% = *N*/819)	Female *N* (% = *N*/1549)	*p* value
Skeletal muscle mass	Low	34 (4.2)	258 (16.7)	≤ 0.001*
	Normal	367 (44.8)	719 (46.4)	
	Over	418 (51)	572 (36.9)	
B confidence interval (L–U)		10.126 (9.646–10.605)		≤ 0.001**
Fat‐free mass	Low	44 (5.4)	227 (14.7)	≤ 0.001*
	Normal	375 (45.8)	600 (38.7)	
	High	400 (48.8)	722 (46.6)	
B confidence interval (L–U)		16.541 (15.749–17.333)		≤ 0.001**
Visceral fat level	Healthy level	431 (52.6)	448 (28.9)	≤ 0.001*
	Risk of excessive levels	388 (47.4)	1101 (71.1)	
B confidence interval (L–U)		−2.994 (−3.595 to −2.392)		≤ 0.001**
Percent body fat	Low	6 (0.7)	2 (0.1)	≤ 0.001*
	Normal	90 (11)	7 (0.5)	
	High	723 (88.3)	1540 (99.4)	
B confidence interval (L–U)		−10.687 (−11.473 to −9.901)		≤ 0.001**

* Chi‐Square.

** Linear Regression, *p*‐value at ≤ 0.05.

Table 4 indicates that around half of both males and females were obese, 49.2% and 57.7%, respectively. Moreover, approximately two‐thirds of the males exhibit a high Basal Metabolic Rate (66.2%); in contrast, most females (89.6%) show a low Basal Metabolic Rate. In relation to patients' body scores, males presented a body score range from normal (32.8%) to low health risk (33.7%), while the largest percentage of females fell within the low health risk (39.7%) to high health risk (43.6%) range. Besides, most of the males and females revealed a high waist‐hip ratio (80.3%) and (86.6%), respectively. Also, the largest percent of patients, males and females, indicated over the range of Obesity Degree (83.9%) and (81.6%), respectively. Furthermore, all the findings are associated with patients' gender, except for the Obesity Degree. Likewise, a linear regression reveals that male gender has a higher impact on body Basal metabolic rate, In body score, and Waist‐hip ratio than females, while showing a lower impact on Body mass index and Obesity degree at the *p*‐value ≤ 0.05.

**Table 4:** Distribution of the patients by their body calculated findings.

		Male *N* (% = *N*/819)	Female *N* (% = *N*/1549)	*p* value
Body mass index	Underweight	11 (1.3)	37 (2.4)	≤ 0.001*
	Normal weight	147 (17.9)	313 (20.2)	
	Overweight	258 (31.5)	306 (19.8)	
	Obesity	403 (49.2)	893 (57.7)	
B confidence interval (L–U)		−1.431 (−2.267 to −0.595)		0.001**
Basal metabolic rate	Low	5 (0.6)	1388 (89.6)	≤ 0.001*
	Normal	272 (32.2)	156 (10.1)	
	High	542 (66.2)	5 (0.3)	
B confidence interval (L–U)		357.271 (340.160–374.383)		≤ 0.001**
In body score	High health risk	231 (28.2)	675 (43.6)	≤ 0.001*
	Low health risk	276 (33.7)	615 (39.7)	
	Normal	269 (32.8)	255 (16.5)	
	Athlete	43 (5.3)	4 (0.3)	
B confidence interval (L–U)		3.727 (2.801–4.653)		≤ 0.001**
Waist‐hip ratio	Low	18 (2.2)	5 (0.3)	≤ 0.001*
	Normal	143 (17.5)	202 (13)	
	High	658 (80.3)	1342 (86.6)	
B confidence interval (L–U)		0.030 (0.023–0.038)		≤ 0.001**
Obesity degree	Below range	28 (3.4)	55 (3.6	0.346*
	Normal range	104 (12.7)	230 (14.8)	
	Over range	687 (83.9)	1264 (81.6)	
B confidence interval (L–U)		−10.111 (−13.973 to −6.248)		≤ 0.001**

* Chi‐Square.

** Linear Regression, *p*‐value at ≤ 0.05.

## 4 Discussion

The study of 2368 patients underscores significant gender differences in body composition, muscle‐fat ratios, and overall health risks, reinforcing the critical role gender plays in medical assessments and interventions. These findings align with existing research while offering new insights into gender‐specific health risks, such as obesity, cardiovascular disease, and metabolic syndrome.

The findings of this study should also be viewed in the context of rising obesity rates in Iraq and the broader Middle East, which have been driven by rapid urbanization, changing dietary patterns, and reduced physical activity levels. Recent data suggest a growing public health burden of obesity‐related conditions in the region [16]. Moreover, several unmeasured confounders, such as socioeconomic status, dietary intake, and physical activity, are known to influence body composition and may have impacted our results. These factors were not included in the current analysis and represent important areas for future research to better understand the multifactorial nature of body composition variability in surgical patients.

### 4.1 Body Composition: Gender‐Specific Differences

The analysis showed that men generally had higher levels of total body water, protein, and minerals than women. It is commonly recognized that morphological traits and body composition parameters are influenced by both exogenous (external environmental variables) and endogenous (internal genetic) influences [17, 18]. Consistent with other studies, the results show that testosterone's anabolic effects cause males to typically have higher total body water, protein, and mineral levels [19, 20]. Recent research, like that of Sanchez et al. [21], has revealed gender differences in muscle preservation and metabolic efficiency. This supports the idea that skeletal muscle mass protects against health conditions associated with obesity. It is important to consider that the gender imbalance in our sample, with a predominance of female participants, may have influenced the comparative findings. Future studies with a more balanced gender distribution are necessary to validate these observations across both sexes.

### 4.2 Muscle‐Fat Analysis: Implications for Health

Compared to female patients, male patients from 2368 had higher levels of skeletal muscle mass and fat‐free mass; conversely, female patients had higher levels of visceral fat and percent body fat, and these results varied according to the patient's gender. Similarly, the study examined the association between middle‐aged persons' lung function and their muscle‐to‐fat (MF) ratio. Men were found to have more skeletal muscle mass and fat‐free mass than women [22]. Other research published in the Journal of Nutrition, Health & Aging, titled “Comparison of skeletal muscle mass to fat‐free mass ratios among different ethnic groups,” delves into the topic of males typically possessing greater skeletal muscle mass and fat‐free mass than women. The study emphasizes how testosterone aids muscle development and preservation [23]. On the other hand, women demonstrated increased fat mass, notably in subcutaneous and visceral areas. Estrogen is the primary cause of this gender‐based disparity in fat distribution [24]; Karastergiou et, al. 2012). Despite effective fat oxidation during exercise, the health risks are worsened by women's propensity to retain fat in visceral areas [25]. Women's hips and thighs, which are vital for reproductive health, might get fattened due to estrogen [26].

### 4.3 Body Metrics and Health Risk: Obesity and Central Fat Distribution

Regarding the body findings, both genders' results indicate high waist–hip ratios and levels of obesity that are above average. Although men have a higher basal metabolic rate, women show a greater risk to their health. However, aside from the level of obesity, the results listed above show a significant association with the patients' gender. A serious public health concern is highlighted by the prevalence of obesity in both sexes, as measured by BMI. However, waist‐hip ratios, also known as risk factors for metabolic and cardiovascular illnesses, were higher in women, indicating a higher degree of abdominal obesity [26]. Even though women had higher body fat percentages, it is interesting to note that men had higher basal metabolic rates (BMR). Because muscular tissue is metabolically active and demands more energy to sustain, males tend to have larger muscle mass [20]; Kelley & Goodpaster, 2022). In the investigation of the biological underpinnings of fat distribution in men and women, Karastergiou et al. [27] observed the different “apple” and “pear” morphologies, with males storing fat around the belly and women around the hips.

### 4.4 Methodological Considerations and Future Directions

To mitigate known limitations of BIA, all measurements in this study were performed preoperatively in a fasting state, prior to fluid administration, and under consistent ambient conditions. The same InBody 270 device and a single trained operator were used throughout to enhance measurement consistency and reduce inter‐observer variability. These steps were essential given BIA's sensitivity to hydration status and its comparatively lower precision than gold‐standard methods such as DEXA.

Nonetheless, even with standardized measurement protocols, the absence of postoperative follow‐up data, such as complication rates, length of stay, or recovery profiles, limits the ability to directly link preoperative body composition to surgical outcomes. This restricts the clinical applicability of our findings. To fully assess the predictive value of preoperative BIA assessments, future research should adopt longitudinal designs that track patients through recovery and explore how body composition influences postoperative course and long‐term outcomes.

## 5 Limitation

This study has several limitations. Its cross‐sectional design restricts causal inference between body composition and surgical outcomes. The use of convenience sampling from a single hospital may limit the generalizability of findings to broader populations. However, it is important to note that Erbil Teaching Hospital is the main referral center in Erbil City for patients with nutrition‐related conditions, as it's the only official Public Nutritional Clinic in the city. Most physicians in the area routinely refer such patients to this hospital. Therefore, while our study is based on a single center, it reflects a significant portion of the patient population with diet‐related diseases in Erbil City.

Additionally, the absence of postoperative follow‐up data and functional outcomes weakens the ability to assess clinical impact. Although BIA is a reliable tool, it may be affected by factors such as hydration status. Furthermore, the gender imbalance in the sample, with more female participants, may have influenced the comparative analysis.

## 6 Conclusion

This study is among the first large‐scale investigations in Iraq to analyze gender‐specific body composition profiles using Bioelectrical Impedance Analysis (BIA) in a surgical patient population. The findings reveal a distinct pattern of body composition differences between males and females, which may have implications for preoperative risk stratification and personalized nutritional support. By highlighting the high prevalence of obesity, abnormal waist‐hip ratios, and imbalanced muscle‐fat distribution across genders, the study provides important baseline data for a Middle Eastern surgical population that is underrepresented in the literature. However, given the cross‐sectional design and the absence of postoperative outcome data, our findings should be interpreted with caution. While body composition differences are strongly associated with gender, causal relationships with surgical risk, recovery, or prognosis cannot be established. Future research should therefore adopt longitudinal designs with postoperative follow‐up to evaluate how these preoperative body composition parameters influence complication rates, recovery, and long‐term outcomes.

## Author Contributions

Alaa Noori Sarkees was responsible for conceptualizing the study and overseeing the project administration. Rondik Khalid Mosa and Sazgar Khald Ibrahim contributed to the development of the methodology, with Rondik Khalid Mosa also conducting the formal analysis. The investigation and data collection were carried out by Sazgar Khald Ibrahim and Salih M.S. Zebari, while Alaa Noori Sarkees managed data curation. The initial draft of the manuscript was written by Sazgar Khald Ibrahim, with subsequent revisions and critical editing performed by Rondik Khalid Mosa and Alaa Noori Sarkees. The study was supervised by Salih M.S. Zebari, ensuring accuracy and rigor in all aspects of the research. All authors have read and approved the final version of the manuscript. Alaa Noori Sarkees had full access to all of the data in this study and takes complete responsibility for the integrity of the data and the accuracy of the data analysis.

## Funding

The authors received no specific funding for this work.

## Ethics Statement

The study protocol was approved by the ethical committee of Hawler Medical University (6) on 8‐5‐2024.

### 1 Consent

All human participants provided informed consent to participate in the study prior to their inclusion.

### 2 Conflicts of Interest

The authors declare no conflicts of interest.

## Transparency Statement

The lead author Alaa Noori Sarkees affirms that this manuscript is an honest, accurate, and transparent account of the study being reported; that no important aspects of the study have been omitted; and that any discrepancies from the study as planned (and, if relevant, registered) have been explained.

## Data Availability

The datasets generated and analyzed during the current study are available from the corresponding author upon reasonable request.
